# Next generation sequencing on patients with LGMD and nonspecific myopathies: Findings associated with *ANO5* mutations

**DOI:** 10.1016/j.nmd.2015.03.011

**Published:** 2015-07

**Authors:** Marco Savarese, Giuseppina Di Fruscio, Giorgio Tasca, Lucia Ruggiero, Sandra Janssens, Jan De Bleecker, Marc Delpech, Olimpia Musumeci, Antonio Toscano, Corrado Angelini, Sabrina Sacconi, Lucio Santoro, Enzo Ricci, Kathleen Claes, Luisa Politano, Vincenzo Nigro

**Affiliations:** a Telethon Institute of Genetics and Medicine, Pozzuoli (NA), Italy; b Dipartimento di Biochimica, Biofisica e Patologia Generale, Seconda Università di Napoli, Napoli, Italy; c Fondazione Don Gnocchi, Italy; d Dipartimento di Neuroscienze e Scienze riproduttive ed odontostomatologiche, Università di Napoli “Federico II”, Napoli, Italy; e Center for Medical Genetics, Ghent University Hospital, Ghent, Belgium; f Department of Neurology, Ghent University Hospital, Ghent, Belgium; g Biochimie et génétique moléculaire, Centre hospitalier Cochin, Paris, France; h Dipartimento di Neuroscienze, Università di Messina, Messina, Italy; i Dipartimento di Neuroscienze, Università di Padova, Padova, Italy; j Centre de Référence Maladies Neuromusculaires – SLA, Hôpital Archet 1, CHU de Nice, Nice, France; k Dipartimento di Medicina Sperimentale, Seconda Università di Napoli, Napoli, Italy

**Keywords:** Next generation sequencing, Muscular dystrophy, LGMD2L, Anoctamin, NGS screening, Targeted resequencing, Limb girdle muscular dystrophy

## Abstract

We studied 786 undiagnosed patients with LGMD or nonspecific myopathic features to investigate the role of ANO5 mutations in limb-girdle muscular dystrophies (LGMDs) and in nonspecific myopathies using the next generation sequencing (NGS) approach. In 160 LGMD patients, we first sequenced hotspot exons 5 and 20 and then sequenced the remaining part of the coding region. Another 626 patients, recruited using broader inclusion criteria, were directly analyzed by targeted NGS. By combining NGS and Sanger sequencing, we identified 33/786 (4%) patients carrying putative pathogenic changes in both alleles and 23 *ANO5* heterozygotes (3%). The phenotypic spectrum is broader than expected, from hyperCKemia to myopathies, with lack of genotype/phenotype correlations. In particular, this is currently the largest screening of the *ANO5* gene. The large number of heterozygotes for damaging mutations suggests that anoctaminopathies should be frequent and often nonpenetrant. We propose the multiple genetic testing by targeted NGS as a first step to analyze patients with nonspecific myopathic presentations. This represents a straightforward approach to overcome the difficulties of clinical heterogeneity of *ANO5* patients, and to test, at the same time, many other genes involved in neuromuscular disorders.

## Highlights

• We have carried out the largest screening of the ANO5 gene.
• We identified 33 patients (4%) with pathogenic changes in both alleles and 23 heterozygotes (3%).
• The identification of a *ANO5* carrier is not to be considered an uncommon finding.
• The anoctaminopathies have an extremely high genetic and phenotypic heterogeneity.
• NGS-based strategies are perfect to dissect the clinical variability in NMDs.

## 1 Introduction

The diagnosis of autosomal recessive limb-girdle muscular dystrophies (LGMDs) is complex for the presence of a number of different conditions with similar clinical presentation [1]. The genetic studies have demonstrated the involvement of at least 23 different genes for the LGMD2 forms [2] and others that are involved in metabolic, congenital or other myopathies that can also present with a clinical LGMD-like phenotype [3]. For the correct diagnosis of specific forms four elements may be of pivotal importance: 1) the clinical picture; 2) the muscle biopsy; 3) the imaging; 4) the DNA results, with the last approach that is changed dramatically in the course of the present study. We have studied one of the most interesting forms of LGMD that is caused by recessive mutations in a gene coding for a calcium-activated chloride channel, known as anoctamin 5 (*ANO5*) [4]. The *ANO5* gene at 11p14.3 spans 90,192 bp and contains 22 exons, the coding sequence is 2.7 kb for 913 amino acids. This form, according to the order of mapping, has been defined as LGMD2L. Genetic studies in some countries have shown that LGMD2L may be a very common form of LGMD [5]. In place of the proximal limb-girdle presentation, some patients show Miyoshi-like muscular dystrophy type 3 (MMD3) [6]. Dominant variations in the same gene have been associated to gnathodiaphyseal dysplasia (GDD) [7].

The LGMD2L phenotype was described for the first time in 2007 in 14 patients of French Canadian origin, showing atrophy and weakness of the quadriceps and biceps brachii muscles [8]. In 2010, *ANO5* was identified as the causative gene [4]. More recently, it has been indicated as the third most common form of LGMD in the North of Europe and the c.191dupA mutation has been shown to be the most prevalent because of a founder effect [5,9].

Distinctive features of LGMD2L versus other LGMD forms are: 1) the sex imbalance, with females that are less frequently or severely affected than males [10]; 2) asymmetry of muscle involvement that is rare among the LGMD and frequent in FSH [4]; 3) the pain following exercise that is typical of metabolic or inflammatory conditions [11].

All the previous studies have evidenced the extreme heterogeneity of the observed phenotypes, comprising the asymmetric atrophy and weakness affecting primarily the quadriceps, hamstrings and biceps, an adulthood onset and a slow progression [4,5,10,12,13]. The weakness of both distal and proximal lower limbs, exercise intolerance, a so called “pseudometabolic” phenotype and also amyloid deposits in the muscles [11,14] are all features present in patients affected by anoctaminopathy or dysferlinopathy [15].

In this paper, we describe the results of a genetic screening in a subset of patients with a broad clinical phenotype of LGMD or generic myopathy. In total, we have fully sequenced the *ANO5* gene in 786 patients using Sanger and/or Next Generation Sequencing (NGS): in this cohort, we have found 33 cases belonging to 28 families. Our data confirm the genetic heterogeneity of the *ANO5* gene and highlight the weak genotype–phenotype correlation.

## 2 Methods

### 2.1 Sample collection

From a large collection of families with a clinical diagnosis of LGMD or with molecularly uncharacterized myopathy, we recruited 786 patients. In particular, 712/786 (90.6%) patients were from Italian families. Additional patients (n = 74) were from Belgium (39), France (8), Finland (4), Brazil (3), Turkey (3), Romania (3), Morocco (3), Germany (2), Russia (2), Greece (1), Israel (1), Uganda (1), Spain (1), Libya (1), Cyprus (1) and The Netherlands (1),

In all the cases, genomic DNA has been tested and, when available, a further analysis on mRNA from blood or from muscle has been performed.

### 2.2 Clinical and diagnostic criteria

Based on literature evidence, as a first step, we screened 160 patients for mutations in exon 5 and in exon 20. Because of the low mutation rate detected in these exons, we extended the analysis to all the other exons.

All the patients recruited for the first step have an LGMD or an LGMD-like phenotype, including a raised serum creatine kinase (CK), progressive muscle weakness affecting primarily the shoulder girdle and pelvic muscles and a muscle biopsy with dystrophic features. All of them show an autosomal recessive inheritance or are sporadic cases. Moreover, most (about 85%) of the 160 samples analyzed by PCR and Sanger sequencing had resulted negative for mutations in *DYSF* and *CAPN3* genes.

Another 626 samples were recruited for NGS, including all the *ANO5* exons and the 10 flanking nucleotides.

The inclusion criteria for the NGS screening were less stringent. Samples of still living patients, affected by an uncharacterized muscular dystrophy (65%) or myopathy (35%), were included. These patients had a wide spectrum of clinical phenotypes, ranging from an isolated hyperCKemia to mild or severe conditions with a variable age of onset and progression. A large portion of these patients (30%) had not been screened previously and less than 20% had been analyzed for mutations in LGMD recessive genes (in particular *CAPN3*, *DYSF* or sarcoglycan genes).

Moreover, DNA samples from 52 unaffected people were sequenced as a control group.

Written informed consent for DNA analysis was obtained from all the recruited patients or their caregivers when primary diagnostic procedures were performed, with explicit consent for future use for research purposes, according to the Declaration of Helsinki. Approval for the study was obtained by the Seconda Università di Napoli Ethics Committee.

### 2.3 Molecular analysis

Genomic DNA was extracted from peripheral blood by phenol/chloroform. All the *ANO5* exons have been amplified by PCR using M13-tailed primers. M13 primers have been used to perform Sanger sequencing using an ABI PRISM 3130 XL automatic DNA Sequencer Genetic Analyzer (Applied Biosystems, Foster City, CA, USA). We used a TRIzol reagent (Invitrogen, Carlsbad, CA, USA) according to the manufacturer's instructions to extract RNA from the muscle biopsies and the PAXgene Blood RNA Kit (Qiagen, Hilden, Germany) to extract RNA from the blood.

The retrotranscription reaction was performed using 2 mg of total mRNA, according to the procedure described in the SuperScript III kit (Invitrogen).

We amplified the *ANO5* cDNA in seven overlapping fragments. Supplementary Table S1 lists primers and PCR conditions.

For NGS screening, samples were enriched using HaloPlex Target Enrichment System (Protocol version D, August 2012, Agilent Technologies, Santa Clara, CA, USA) [16].

For each of the novel mutations identified, amino acid change, presence in dbSNP v137 [17], frequency in NHLBI Exome Variant Server (http://evs.gs.washington.edu/EVS) and 1000 genomes large scale projects (http://www.1000genomes.org) [18], conservation and causative effects, using different prediction algorithms [19–21], were evaluated.

To assess intronic and exonic mutations leading to splicing defects, a free bioinformatic tool (http://www.fruitfly.org/seq_tools/splice.html) [22] was consulted.

## 3 Results

To study 160 patients with undiagnosed LGMD, we first sequenced the hotspot regions at exons 5 and 20 of the *ANO5* gene, but the screening was diagnostic in two families only (I and XVI, homozygotes for c.191dupA and c.2272 C > T, respectively). Ten additional patients were heterozygous for a single *ANO5* mutation. We next extended the analysis to all the other exons by PCR and Sanger sequencing. By this exon-by-exon scanning, we were able to detect another fourteen mutations, concluding the genetic diagnosis in other 15 patients (Table 1).

To profit from the extraordinary throughput of next generation sequencing [23], we included the *ANO5* gene in a large and accurate screening of genes causing neuromuscular disorders [16]. In particular, the *ANO5* exons and the ten flanking bases were >90% covered at no less than 100× (Supplementary Fig. S1) after a Haloplex-based enrichment. We studied further 626 patients with broader phenotypic presentation, ranging from classic LGMD phenotype to congenital myopathies, nonspecific myopathic features or hyperCKemia. In this way, we identified other 15 *ANO5* patients. All the variants identified by NGS were then confirmed by the dideoxy method. Interestingly, we detected different *ANO5* variants in both alleles in patients XVIII and XXIV. These were classified as affected by a congenital myopathy with a hypothesized dominant transmission. However, they did not show mutations in the other genes causing congenital myopathies. Considering their specific phenotype and the unavailability of other relatives' samples to study the status and the segregation of their mutations, we were not able to correctly interpret them.

We also identified 23 patients with a single mutated allele, including one (XXXVIII) with two mutations *in cis* on the same chromosome.

To avoid the risk to miss mutations, the DNA samples from heterozygous patients were also resequenced exon-by-exon and no additional variations were detected. Array-CGH (Motorchip [24]) testing was also negative.

Finally, three normal control samples also showed heterozygous variants in *ANO5*, including a novel missense substitution (ctrl1).

By combining NGS and Sanger sequencing, we identified 33/786 individuals, from 28 different families, homozygous or compound heterozygous for mutations in the *ANO5* gene. Forty-three mutations were detected in 16/22 exons. Twelve of them had already been detected and described in literature; on the contrary, thirty-one had never been described previously (Table 1).

### 3.1 Novel mutations

#### 3.1.1 Missense and nonsense mutations

Twenty-four novel missense and nonsense mutations were detected. In particular, four of them (c.1207 C > T, c.1213 C > T, c.1261 C > T and c.1639 C > T) introduced a premature stop codon. All the other variants determined an amino acidic change and their clinical significance was evaluated by different bioinformatic tools.

The mutations identified in homozygosity (c.161 T > C in II and c.2498 T > A in VII) or in compound heterozygosity (c.2489 C > T in XV, c.2342 in XLV and c.2411C > G in XLII) were all predicted to be causative by at least two out of three tools.

#### 3.1.2 Point mutations modifying a splicing site consensus

**Fig. 1:**
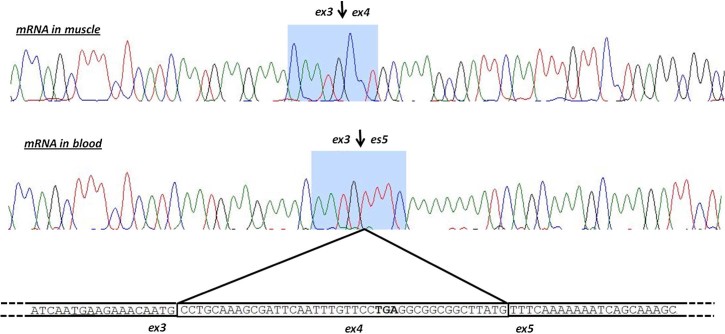
Alternative splicing of exon 4. *ANO5* mRNA in muscle shows the full-length isoform containing the exon 4 (A). Blood isoform is differentially spliced, the exon 4 is removed and the exon 3 is directly joined with the exon 5 (B).

Four point mutations (c.294 G > A; c.649-2 A > G; c.1119 + 1 G > A and c.2235 + 1 G > A) were expected to modify a splicing site, as suggested by bioinformatic tools. We analyzed *ANO5* mRNA in the leukocytes (for patient XXII) or in the muscle (V), to confirm the splicing effect. As detected on control samples, the leukocyte *ANO5* isoform lacks exon 4, but maintains the reading frame and determines a predicted protein 14 amino acids smaller (Fig. 1).

**Fig. 2:**
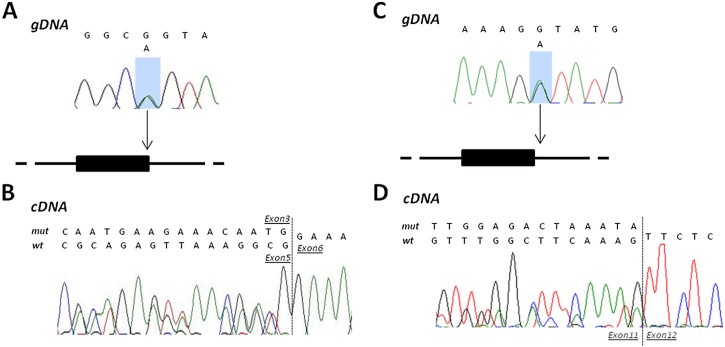
Intronic mutations affecting the splicing in patients XIX and V. In patient XIX, the G > A mutation in the last exonic nucleotide (A) causes the loss of the canonical splicing site (B). In blood cDNA, the sequence shows the normal splicing, which connects exon 3 and exon 5, and the abnormal splicing with the complete loss of exon 4. In patient V, the G > A mutation in the first intronic nucleotide (C) causes the activation of a cryptic splicing site twenty nucleotides upstream (D), as evidenced on muscular cDNA. The loss of the last twenty nucleotides of exon 11 produces a frame-shift and a premature stop codon five amino acids later.

In patient XXII, the mutation produces a shortened mRNA without exons 4 and 5 (Fig. 2A, B). The extra-skipping of exon 5 causes an in-frame deletion of 114 nucleotides encoding 38 amino acids. However, both DNA and RNA analyses did not allow us to detect a second mutation in this patient that remains formally undiagnosed.

The muscular mRNA of patient V revealed the activation of a cryptic splice site 20 nucleotides upstream the natural 3′ end of exon 11 (Fig. 2C–D). The frame-shift results in a premature stop codon after five amino acids. For splice site mutations in patients XIX and XX, we did not study muscle mRNA, but there is little doubt about their deleterious effect.

#### 3.1.3 Small deletions and insertions

We found two small deletions in the same patient (VI). In particular, in exon 6 we detected a c. 304–308 delAAAGA, causing a frame-shift with the substitution of a Lysine with a Valine 102 and a premature stop codon after a single amino acidic residue.

In exon 19, we found a 3-nucleotide deletion c. 2102–2105 delATA, causing the loss of an Asparagine 701. This residue, evolutionarily conserved, is the first amino acid of the putative sixth cytoplasmatic loop and its loss is predicted to be damaging.

An insertion of a single nucleotide was found in one allele of patient XLVII and it determined an immediate premature stop codon.

### 3.2 Phenotypic spectrum of *ANO5* patients

As already reported [10], also in our cohort of patients, the number of affected males (21 = 64%) was higher than that of females (12 = 36%). The age of onset varied significantly (mean = 26.81; min = 13; max = 44). Interestingly, four *ANO5* patients (IV,1; IV,2; X,2; XIV,2) are still asymptomatic even if they showed increased CK serum levels. The average CK values were 3200 IU/l ranging from 500 IU/l to 9800 IU/l.

The clinical signs of patients with LGMD2L presentation, such as the early asymmetric quadriceps weakness, the high CK and the slow progression, and the histological features, including mild myopathic changes, were in agreement with literature data (Table 2) [13,25–27]. All the patients are still ambulant, but two that occasionally use walking aids (aged 71 and 75).

When assessed, cardiac and respiratory functions were normal with the exception of patient V, who showed a short PQ interval, and of patient XII, who is suffering from a restrictive respiratory insufficiency.

For 15 families, a brief summary of the phenotype was added as Supplementary appendix. Patients XV, XVI and XVII have been previously characterized elsewhere [13].

## 4 Discussion

By combining NGS and Sanger sequencing, we have carried out the largest screening of the *ANO5* gene in 786 myopathic patients and 52 controls. In our cohort of patients, thirty-three are homozygous or compound heterozygous for causative mutations in *ANO5*. Interestingly only 18/33 are Italian (although they are 90% of the cohort), providing a further evidence of lower frequency of anoctaminopathies in this country [12] where dysferlinopathies and calpainopathies still remain the most common form of LGMD [28]. In contrast, we have evidenced a single heterozygous variant in 3% (23/786) of patients. Some of these may be non-pathological rare variants, but others, such as c.191dupA, are well-known causative mutations. When fully studied, heterozygous patients show no hidden mutation on the second allele. Is this compatible with the disease prevalence? Previous published papers have evidenced the high prevalence of anoctaminopathies in Northern Europe: in particular, a prevalence of 0.27/100,000 has been estimated in the North of England [5] and of 2/100,000 in the Finnish population [13]. However, a rarer frequency of variants has been reported elsewhere [12].

To explain 3% of heterozygotes, we propose two hypotheses:

1 We would find a similar number of heterozygotes in any other cohort of subjects, because the frequency of pathological alleles is at least 10-fold higher than expected (>0.01 instead of 0.001). This immediately indicates that over 90% of cases with both *ANO5* mutations should be quite healthy, in the absence of a second unknown hit.
2 We have found many heterozygous subjects, because they are true patients: this suggests that *ANO5*-myopathy could be transmitted as a dominant trait, in the presence of a second unknown hit. A point in favor of the first hypothesis is the long list of *ANO5* variants present in the Exome Variant Server and in dbSNP: *ANO5* is certainly a highly polymorphic gene. In fact, eighty variants with a frequency lower than 1.5% are listed in EVS (Supplementary Table S2) for a total of 959 carriers; 20 subjects (0.3%) are heterozygous for the well-known c.191 dupA and 159 (2.4%) show a putative damaging variant (total 3%). The identification of a carrier of a single mutation in *ANO5* gene is not to be considered an uncommon finding and it will be important to identify a second putative hit. Since deleterious copy number imbalances have been estimated in 5–10% of patients affected by neuromuscular disorders [24], copy number variants involving noncoding regulatory regions [29,30] could affect the *ANO5* expression in some tissues. Other explanations involve mutations in other genes belonging to the same pathway, the effect of modifier genes, epigenetic changes or environmental factors.

Interestingly, the phenotype–genotype analysis shows the absence of a correlation. All the asymptomatic patients have increased creatine kinase levels, supporting the hypothesis of the variable expressivity of *ANO5* myopathy. In our cases, the expressivity seems to be independent of causative mutations and also unrelated to sex and age. In particular, the lack of a clear genotype–phenotype correlation is evident either comparing different families or even focusing on different patients within the same family (for example, the patient X,2, still asymptomatic, has a younger brother presenting with cramps and myalgia). The interfamilial variability could reflect a specific genetic background and the putative presence of a second hit, as postulated. On the contrary, the intra-familial heterogeneity could be due to different external factors, including lifestyle, sport activity and diet, which should be further investigated.

**Fig. 3:**
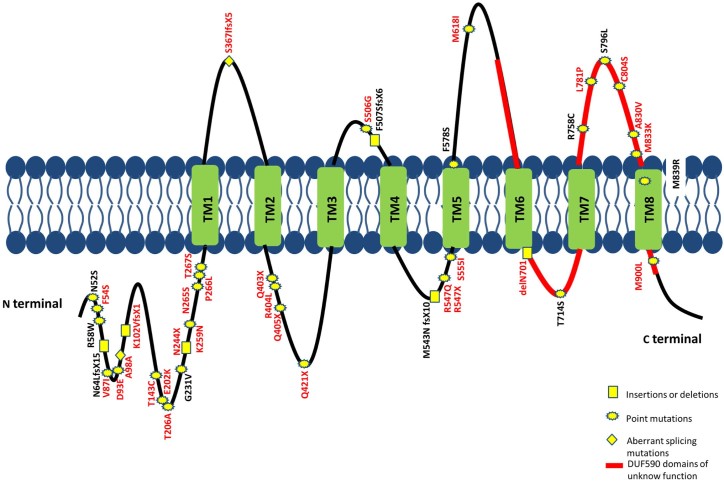
Locations of *ANO5* mutations. Black label: previously reported recessive mutations. Red label: novel mutations reported in this study. Splice mutations for which their effect on the mRNA has not been verified have been omitted.

The anoctaminopathies are also characterized by an extremely high genetic heterogeneity [10,25]. Interestingly, among our patients with a complete molecular diagnosis of anoctaminopathy, c.191dupA and c.2272C > T, the most common variation described so far, represent less than 30% of identified mutations and they both account for only 20% of those detected in the Italian patients. On the contrary, we have identified 31 novel variants, confirming the genetic heterogeneity of *ANO5* and demonstrating that the simple screening of one or two recurrent mutations cannot be considered effective in Southern European populations (Fig. 3).

Considering the power of next generation sequencing [23] and the clinical and genetic variability of muscular dystrophies, diagnostic approaches based on NGS are becoming increasingly frequent [31]. However, for clinical and diagnostic aims, a targeted resequencing of genes already known to be linked to the specific pathological condition is advisable [32] and *ANO5* has to be included. Our data also demonstrate the utility of this approach, highlighting, however, how important the correct interpretation of the data generated by these approaches could be. Moreover, these NGS-based strategies are perfect to dissect the clinical variability [33], meeting, in this way, with the next challenges of research.

In conclusion, we suggest that the terms “anoctaminopathy” or “*ANO5* myopathy” better define a heterogeneous disease caused by mutations in the *ANO5* gene, irrespective of the first proximal (LGMD2L), distal symptoms (Miyoshi myopathy) or characterized by other myopathic features.

## Figures and Tables

**Table 1:** List of patients and controls with ANO5 variants.

Sample ID	Status	Mutations
I,1^s^	hom	c.191 dupA (exon 5) p.Asn64LysfsX15^6^
I,2^f^	hom	c.191 dupA (exon 5) p.Asn64LysfsX15^6^
II^s^	hom	**c.161T > C (exon 4) p.Phe54Ser**
III^s^	hom	c.172C > T (exon 4) p.Arg58Trp^31^
IV,1^s^	c.het	c.1733T > C (exon 16) p.Phe578Ser^8^ + c.2272C > T (exon 20) p.Arg758Cys^6^
IV,2^f^	c.het	c.1733T > C (exon 16) p.Phe578Ser^8^ + c.2272C > T (exon 20) p.Arg758Cys^6^
V^s^	c.het	**c.1119** **+** **1 G** **>** **A (exon 11) p.Ser367IlefsX5** + c.2272C > T (exon 20) p.Arg758Cys^6^
VI^s^	c.het	**c.304–308 delAAAGA (exon 6) p.Lys102ValfsX1** **+** **c.2102–2105 delATA (exon 19) p.ΔAsn701**
VII^s^	hom	**c.2498T > A (exon 21) p. Met833Lys**
VIII^s^	hom	**c.1639C > T (exon 16) p.Arg547X**
IX,1^s^	c.het	c.191 dupA (exon 5) p.Asn64LysfsX15^6^ + c.1733T > C (exon 16) p. Phe578Ser^8^
IX,2^f^	c.het	c.191 dupA (exon 5) p.Asn64LysfsX15^6^ + c.1733T > C (exon 16) p. Phe578Ser^8^
X,1^s^	c.het	c.191 dupA (exon 5) p.Asn64LysfsX15^6^ + c.2516T > G (exon 21) p.Met839Arg^16^
X,2^f^	c.het	c.191 dupA (exon 5) p.Asn64LysfsX15^6^ + c.2516T > G (exon 21) p.Met839Arg^16^
XI^s^	c.het	c.191 dupA (exon 5) p.Asn64LysfsX15^6^ + **c.1261C > T (exon 13) p.Gln421X**
XII^ngs^	hom	c.692G > T (exon 8) p.Gly231Val^6^
XIII^ngs^	hom	c.191 dupA (exon 5) p.Asn64LysfsX15^6^
XIV,1^ngs^	hom	c.1627dupA (exon 15) p.Met543Asn fsX10^17^
XIV,2^f^	hom	c.1627dupA (exon 15) p.Met543Asn fsX10^17^
XV^s^	c.het	c.191 dupA (exon 5) p.Asn64LysfsX15^6^ + **c.2489C > T (exon 21) p.Ala830Val**
XVI^s^	hom	c.2272C > T (exon 20) p.Arg758Cys^6^
XVII^s^	c.het	c.1520 delT (exon 15) p.Phe507SerfsX6^17^ + c.1898-4A > G (exon 18) spl.?^17^
XVIII^ngs^	het/c.het	**c.616A > G (exon 7) p.Thr206Ala** **+** **c.1211G > T (exon 13) p.Arg404Leu**
XIX^ngs^	hom	**c.2235** **+** **1G > A (exon19) spl.?**
XX^ngs^	hom	**c.649-2A > G (exon8) spl.?**
XXI^ngs^	c.het	c.191 dupA (exon 5) p.Asn64LysfsX15^6^ + **c.1213C > T (exon 13) p.Gln405X**
XXII^s^	het	**c.294G > A(exon 5) p.Ala98Ala spl.?**
XXIII^s^	het	**c.1640G > A (exon 16) p.Arg547Gln**
XXIV^ngs^	het/c.het	c.191 dupA (exon 5) p.Asn64LysfsX15^6^ + c.2387C > T (exon20) p.Ser796Leu^32^
XXV^ngs^	het	**c.2698A > C (exon 22) p.Met900Leu**
XXVI^ngs^	het	**c.279C > G (exon 5) p.Asp93Glu**
XXVII^ngs^	het	**c.797 C** **>** **T (exon 9) p.Pro266Leu**
XXVIII^ngs^	het	**c.428A > G (exon 7) p.Tyr143Cys**
XXIX^ngs^	het	**c.777G > T (exon 9) p.Lys259Asn**
XXX^ngs^	het	c.2141C > G (exon 19) p.Thr714Ser^17^
XXXI^ngs^	het	**c.294G > A(exon 5) p.Ala98Ala spl.?**
XXXII^ngs^	het	c.2141C > G (exon 19) p.Thr714Ser^17^
XXXIII^ngs^	het	**c.604G > A (exon 7) p.Glu202Lys**
XXXIV^ngs^	het	**c.800C > G (exon 9) p.Thr267Ser**
XXXV^ngs^	het	c.692G > T (exon 8) p.Gly231Val^6^
XXXVI^ngs^	het	c.155A > G (exon4) p.Asn52Ser^32^
XXXVII^ngs^	het	**c.1664G > T (exon 16) p.Ser555Ile**
XXXVIII^ngs^	het	**c.259G > A p.Val87Ile (exon 5)** + c.692G > T (exon 8) p.Gly231Val^6^ [in cis]
XXXIX^ngs^	het	c.2387C > T (exon20) p.Ser796Leu^32^
XL^ngs^	het	c.191 dupA (exon 5) p.Asn64LysfsX15^6^
XLI^ngs^	het	**c.794A > G (exon9) p.Asn265Ser**
XLII^ngs^	c.het	**c.1207C > T (exon 13) p.Gln403X** **+** **c.2411C > G (exon 20) p.Cys804Ser**
XLIII^ngs^	c.het	c.191 dupA (exon 5) p.Asn64LysfsX15^6^ + c.1520 delT (exon 15) p.Phe507SerfsX6^17^
XLIV^ngs^	hom	c.191 dupA (exon 5) p.Asn64LysfsX15^6^
XLV^ngs^	c.het	c.692G > T (exon 8) p.Gly231Val^6^ + **c.2342T > C (exon 20) p.Leu781Pro**
XLVI^ngs^	c.het	**c.1213C > T (exon 13) p.Gln405X** + c.2387C > T (exon20) p.Ser796Leu^32^
XLVII^ngs^	c.het	**c.729_730insT (exon 8) p.Asn244X** + c.2387C > T (exon20) p.Ser796Leu^32^
XLVIII^ngs^	het	c.155A > G (exon4) p.Asn52Ser^32^
XLIX^ngs^	het	**c.1516A > G (exon15) p.Ser506Gly**
L^ngs^	het	c.2141C > G (exon 19) p.Thr714Ser^17^
LI^ngs^	het	c.2516T > G (exon 21) p.Met839Arg^16^
ctrl1^ngs^	het	**c.1854G > C (exon 17) p.Met618Ile**
ctrl2^ngs^	het	c.2387C > T (exon20) p.Ser796Leu^32^
ctrl3^ngs^	het	c.2387C > T (exon20) p.Ser796Leu^32^

s = Sanger sequencing; f = familial; ngs = NGS analysis; hom = homozygous; het = heterozygous; c.het = compound heterozygous. Novel mutations are in bold.

**Table 2:** Clinical features.

Patient ID	Sex	Current age	Age of onset	CK, IU/l, range	ECG and ultrasound	Spirometry	Clinical phenotype	Biopsy	Loss of walking	Genotype
I,1	M	63	37	2800–9800	Normal	NA	Hyper-Ck-emia, lower quadriceps pain and later mild weakness	Muscular dystrophy with regenerating and necrotic fibers, variation in fibers size (small and hypertrophic)	No	c.191 dupA; p.Asn64LysfsX15 + c.191 dupA; p.Asn64LysfsX15
I,2	F	35	32	2000–3000	Normal	NA	Hyper-CK-emia, followed by lower quadriceps pain doing stairs	NA	No	
II	M	51	36	3000–7000	NA	NA	Weakness of ankle plantar flexors, hamstrings and quadriceps, hypotrophy	Mild dystrophic changes	No	c.161T > C; p.Phe54Ser + c.161T > C; p.Phe54Ser
III	M	31	16	1000–2000	Normal	Normal	Hypertrophy of calves, cramps and fatigability	Sparse rounded hypotrophic fibers and some splitting fibers	No	c.172C > T; p.Arg58Trp + c.172C > T; p.Arg58Trp
IV,1	M	31	19	>5000	Normal	Normal	Hyper-Ck-emia, still asymptomatic	NA	No	c.1733T > C; p.Phe578Ser + c.2272C > T; p.Arg758Cys
IV,2	F	20	15	1000–2000	Normal	Normal	Hyper-Ck-emia, still asymptomatic	NA	No	
V	F	39	24	3000–4000	PQ short	Normal	Distal weakness arm, hyper-CK-emia, myalgia, painful contractures	Mitochondrial myopathy	No	c.1119 + 1 G > A; p.Ser367IlefsX5 + c.2272C > T; p.Arg758Cys
VI	F	44	39	3000–4000	Normal	Normal	Absence of weakness, mild calf hypotrophy	Central nuclei and increased fiber size	No	c.304–308 delAAAGA; p.Lys102ValfsX1 + c.2102–2105 delATA; p.ΔAsn701
VII	M	46	17	3500–8000	Normal	Normal	Weakness of ankle dorsal and plantar flexors, hamstrings and adductors	Mild dystrophic changes	No	c.2498T > A; p. Met833Lys + c.2498T > A; p. Met833Lys
VIII	M	75	37	500–2500	Normal	Normal	Symmetric proximo-distal lower limb weakness, quadriceps and calf atrophy, abdominal and neck flexors muscles weakness and dysphonia	NA	No (walk with canes)	c.1639C > T; p.Arg547X + c.1639C > T; p.Arg547X
XVIII	F	57	30	1000	NA	NA	Hyper-Ck-emia, lower quadriceps pain, possible congenital myopathy	Dystrophic changes	No (difficulties in deambulation)	c.616A > G; p.Thr206Ala + c.1211G > T; p.Arg404Leu
IX,1	M	33	17	1600–8700	Normal	NA	Mild shoulder girdle weakness and atrophy, moderate scapular winging, mild pectoral muscle atrophy, mild facial weakness, global mildly decreased muscle mass limbs and trunk	Mild dystrophy, splitting fibers, slight increase in internal nuclei, slight type I fiber predominance	No	c.191 dupA; p.Asn64LysfsX15 + c.1733T > C; p. Phe578Ser
IX,2	M	29	20	1000–2300	Normal	NA	Mild shoulder girdle weakness w/o atrophy or scapular winging, mild pectoral muscle atrophy, mild dorsal and volar forearm muscle atrophy and mild facial weakness	Moderate increase in fibers with central nuclei	No	
X,1	M	47	28	>3000	Normal	Normal	Hyper-Ck-emia, cramps, myalgia	Mild fiber size variability	No	c.191 dupA; p.Asn64LysfsX15 + c.2516T > G; p.Met839Arg
X,2	F	50	NA	500–1500	NA	NA	Hyper-Ck-emia, still asymptomatic	NA	No	
XI	M	44	30	2200–3000	Normal	Normal	Progressive atrophy and weakness of biceps brachii muscles, hamstrings and hip adductors	Myopathic changes and necrotic fibers	No (difficulties in climbing stairs)	c.191 dupA; p.Asn64LysfsX15 + c.1261C > T; p.Gln421X
XII	M	71	32	2700–7800	Normal	FVC = 60%, lying: 50%, use of BIPap	Symmetric proximal UL and proximo-distal LL weakness with quadriceps and calf atrophy , abdominal and neck flexors muscles weakness and swallowing problems	Mild dystrophic changes	No (but sporadic use of wheelchair)	c.692G > T; p.Gly231Val + c.692G > T; p.Gly231Val
XIII	M	38	32	1500–3000	Normal	Normal	Diffuse myalgia, fatigability, absence of weakness and mild unilateral calf hypotrophy.	Minimal changes with increased fiber variability	No	c.191 dupA; p.Asn64LysfsX15 + c.191 dupA; p.Asn64LysfsX15
XIV,1	M	45	40	>1000	Normal	NA	Myalgia and cramps	Mild dystrophic changes	No	c.1627dupA; p.Met543Asn fsX10 + c.1627dupA; p.Met543Asn fsX10
XIV,2	M	38	NA	>1000	Normal	NA	Hyper-Ck-emia, still asymptomatic	NA	No	
XIX	M	54	44	2000–3000	NA	NA	easy fatigability and difficulty walking	Dystrophic features with phagocytosed fibers	No	c.2235 + 1G > A spl. + c.2235 + 1G > A spl.
XX	M	42	15	4800–6600	NA	NA	Hyper-Ck-emia; high-arched feet	Necrotizing myopathy	No	c.649-2A > G spl. + c.649-2A > G spl.
XXI	M	35	29	2830	NA	NA	Hyper-Ck-emia	Slight dystrophy	No	c.191 dupA; p.Asn64LysfsX15 + c.1213C > T; p.Gln405X
XXIV	F	56	47	536	NA	NA	Autosomal dominant myopathy	Myopathic changes	NA	c.191 dupA; p.Asn64LysfsX15 + c.2387C > T; p.Ser796Leu
XLII	F	37	18	>1000	Normal	Normal	LGMD	Myopathic pattern	No	c.1207C > T; p.Gln403X + c.2411C > G; p.Cys804Ser
XLIII	F	23	13	>1000	Normal	Normal	LGMD	Myopathic pattern	No	c.191 dupA; p.Asn64LysfsX15 + c.1520 delT p.Phe507SerfsX6
XLIV	F	33	NA	NA	NA	NA	LGMD	NA	NA	c.191 dupA; p.Asn64LysfsX15 + c.191 dupA; p.Asn64LysfsX15
XLV	F	42	NA	NA	NA	NA	LGMD	NA	NA	c.692G > T; p.Gly231Val + c.2342T > C; p.Leu781Pro
XLVI	M	39	NA	NA	NA	NA	NA	NA	NA	c.1213C > T; p.Gln405X + c.2387C > T; p.Ser796Leu
XLVII	F	NA	NA	NA	NA	NA	NA	NA	NA	c.729_730insT; p.Asn244X + c.2387C > T; p.Ser796Leu

Patients XV, XVI and XVII have been described elsewhere ([16]) and are not included in the table. For patients XLIV–XLVII, detailed clinical data were not available.

## Appendix Supplementary material

Fig S1 Supplementary Figure 1 shows the average sequencing coverage of ANO5 gene.

Table S1 Supplementary Tables e1 contains the list of PCR and RT-PCR primers.

Table S2 Supplementary Table e2 enumerates all the rare ANO5 variants of unknown significance.

Appendix S1 Supplementary text describes the clinical features of ANO5 patients
