# Supplementary material for: Next generation sequencing on patients with LGMD and nonspecific myopathies: Findings associated with ANO5 mutations
Source: Neuromuscul Disord. 2015 Jul;25(7):533–41. doi: 10.1016/j.nmd.2015.03.011 (PMC4502439; doi:10.1016/j.nmd.2015.03.011)
Supplement: Fig S1 — Supplementary Figure 1 shows the average sequencing coverage of ANO5 gene. [file mmc1.pdf]

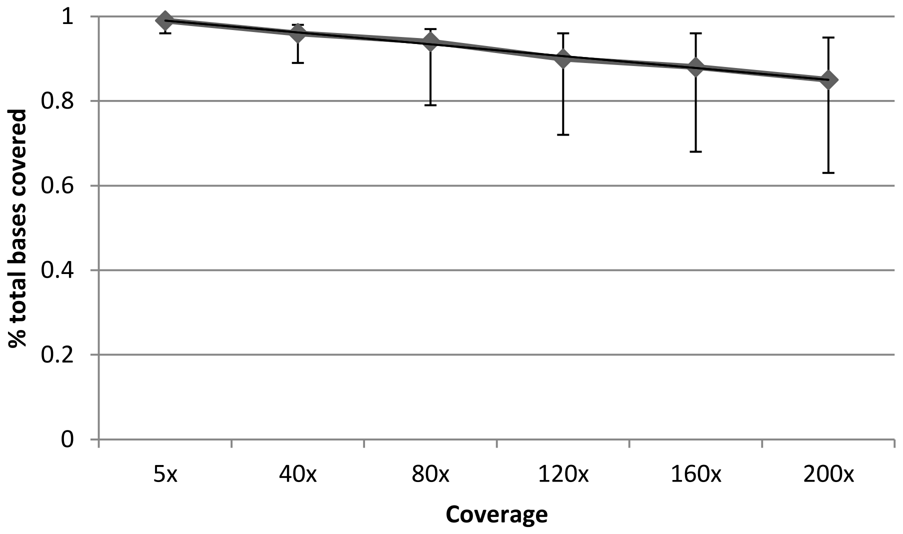

Supplementary Figure 1: ANO5 coverage after target enrichment and sequencing on HiSeq2000. For each sample sequenced, 90% of the ANO5 gene was covered by more than 100 reads.
