# Supplementary material for: Next generation sequencing on patients with LGMD and nonspecific myopathies: Findings associated with ANO5 mutations
Source: Neuromuscul Disord. 2015 Jul;25(7):533–41. doi: 10.1016/j.nmd.2015.03.011 (PMC4502439; doi:10.1016/j.nmd.2015.03.011)
Supplement: Appendix S1 — Supplementary text describes the clinical features of ANO5 patients [file mmc4.docx]

**Supplementary Information**

Family I

The Belgian patient I,1 presented at age 37 with increased CK levels (>3000 U/l) without further complaints or clinical abnormalities at that time. He gradually developed pain and exercise intolerance in both quadriceps muscles, with a mild weakness becoming apparent at around 47 years of age. Clinical examination showed mild shoulder girdle atrophy without any weakness, and a slight atrophy of the quads. He had no cardiac or respiratory abnormalities, and is still ambulant at age 63. A quadriceps muscle biopsy at age 43 showed a dystrophic pattern. Immunohistochemical stains for sarcolemmal and other proteins were normal, as was Western blotting for dystrophin and dysferlin. No written reports about previous molecular tests were shown by the proband. The analysis of exons 5 and 20 evidenced the presence of the homozygous c.191 dupA.

His daughter (I,2), having the same duplication in homozygosis, had CK checked at age 32, and it was increased to about 2400 U/l. At first, she had no symptoms or signs, but the following few years she developed quadriceps pain on exertion. Her mother, who was asymptomatic with normal CK, turned out to have a heterozygous c.191 dupA in the ANO5 gene.

Family II

The proband, an Italian 51-year-old male patient, has been showing hyperCKemia (maximum 7000 UI/l) since he was 30 years old. At age 36 he started to have walking difficulties due to a left leg weakness, followed by the involvement of the contralateral lower limb. Biopsy showed mild dystrophic changes, consisting of a variation in fiber size and the presence of necrotic and regenerating fibers.

By immunohistochemistry and western blot analysis, no significative alterations in the expression of dystrophin, alpha- and gamma-sarcoglycan, merosin, calpain and dysferlin were detected. Previous genetic analyses had excluded mutations in CAPN3, DYSF, FKRP, LMNA and TCAP.

Our screening on the ANO5 gene revealed a novel homozygous missense mutation p.Phe54Ser (c.161 T>C) in exon 4.

Family III

The proband, a 31 year old male Italian patient, has been suffering from cramps and excessive fatigability since he was a teenager and his level of creatine kinases have been constantly high (1000-2000 UI/l). On physical examination, he manifested hypertrophy of the calves. Electromyography (EMG) evidenced slight myopatic changes. Sparse rounded hypotrophic fibers and some splitting fibers were also detected on muscle biopsy, while dystrophin, sarcoglycan, caveolin, dysferlin and spectrin expression was normal. MRI detected no significant changes in the leg muscles. Cardiac and respiratory functions were normal.

Previous tests excluded mutations in CAPN3. ANO5 mutation analysis showed a novel homozygous mutation p.Arg58Trp (c.172 C>T) in exon 4. This change was considered possibly damaging by Polyphen.

Family IV

The proband IV,1 is an Italian male patient investigated for the presence of increased CK levels (>5000 UI/L), not associated with muscle symptoms, observed at the age of 19 years. A family screening for muscle disease revealed that also the younger sister (IV, 2) presented with high CK levels, but not to the same degree. The heart and respiratory function were normal. At the current age of 31 and 20 years respectively, they remain asymptomatic.

Previous molecular tests, performed on patient IV,1, had not detected any mutations in CAPN3, DYSF, TCAP and LMNA genes. Our first screening of exons 5 and 20 evidenced a missense mutation p.Arg758Cys (c.2272 C>T) in exon 20 and an additional variant p.Phe578Ser (c.1733 T>C) in exon 16 were detected by sequencing the whole gene. The same variants were present in IV,2.

Family V

The patient is a 39 year old female of Ukrainian ethnic origin, suffering from myalgia and painful contractures since the age of 24 years, associated with high serum creatine kinase levels (3177 UI/L). Weakness of iliopsoas, quadriceps and tibialis anterior (MRC scale 4) was observed and a mild muscle impairment at lower limbs (left lateralis vastus and right calf) detected by electromyography. Muscle biopsy showed a pattern suggesting a mitochondrial myopathy. A short PQ was seen by ECG; echocolordoppler cardiography was normal. Her son, carrying the mutation c.1119+1 G>A, complains of cramps and shows a mild increase in CK levels. Previous tests evidenced no variations in CAPN3 gene and a heterozygous variant in the exon 6 of DYSF gene (c.509 C>A A170E), with a unknown clinical significance. This variation, in fact, is present in dbSNP with an allelic frequency of 0.959%. ANO5 analyses evidenced a compound heterozygosity for a novel splicing mutation (c.1119+1 G>A) and for the well-known mutation p.Arg758Cys (c.2272 C>T).

Family VI

The patient, a 44-year-old Italian female patient, showed elevated C.K. levels (maximum 3000-4000 UI/l) at age 39 (in 2007). On physical examination only a mild calf hypotrophy was detected in the absence of weakness. Electrocardiogram (ECG), ultrasound and sprirometry, performed to assess cardiac and respiratory function, excluded any cardiac or pulmonary involvement. Electromyography (EMG) showed a pattern compatible with myopathy and muscle biopsy disclosed myopathic changes, such as central nuclei and increased fiber size variability. MRI of the lower limbs showed a replacement of gastrocnemius medialis and soleus. Immunohistochemistry showed a normal expression of dystrophin, alpha- and gamma-sarcoglycan, caveolin, merosin and alpha-dystroglycan, Western blot analysis of calpain and dysferlin was normal. DYSF mutations had been previously excluded.

ANO5 mutation analysis revealed the two small deletions discussed above (c. 304-308 delAAAGA and c. 2012-2015 delATA). Her parents are unaffected and in particular her mother bears the heterozygous deletion in exon 6 and her father the heterozygous deletion in exon 19.

Family VII

The patient, a 46 year-old Italian male, presented at age 17 with a progressive atrophy and weakness of calf muscles. At his most recent examination in February 2012, he showed weakness of ankle extensors and flexors (tibialis anterior and peroneal MRC 1, gastrocnemii MRC 2), hamstrings and adductors (MRC 3). His upper limbs were normal except for a mild extensor digitorum communis weakness. Cardiac and respiratory function were normal too. Serum C.K. levels were high (maximum 8200 UI/l). Electromyography (EMG) and muscle biopsy showed dystrophic changes, and expression of dystrophin, alpha- and gamma-sarcoglycan and merosin was normal on immunohistochemistry.

MRI detected fatty replacement of gastrocnemius medialis and lateralis, soleus, peroneal muscles and, to a lesser extent, tibialis anterior at the leg level. At the thigh level, the involvement was also diffuse with relative sparing of rectus femoris, sartorius, gracilis (asymmetric) and semitendinosus (asymmetric). At the pelvis level, the most affected muscle was the gluteus minimus. T2-STIR sequences showed areas of abnormal signal. Previous analyses excluded significant mutations in the FKRP and DYSF genes. ANO5 mutation analysis revealed a novel homozygous missense mutation p.Met833Lys (c.2498 T>A) in exon 21.

Family VIII

The patient, a 75 year old French man, developed at the age of 37 exercise intolerance and hyperCKemia. At the age of 40, he experienced lower leg weakness and atrophy predominant in the triceps suralis muscles. The weakness has slowly progressed and now involves proximal muscles (predominantly quadriceps and iliopsoas), abdominal and axial muscles. The patient uses a cane to walk and needs assistance for daily life activities. No cardiac or respiratory involvement has been noted. Mild dysphonia has been reported by the speech therapist not associated to swallowing problems.

No written reports about previous molecular tests were shown by the proband. ANO5 analysis evidenced a homozygous point mutation (c.1639 C>T), introducing a premature stop codon in exon 16 (p.Arg547X).

Family IX

The proband (IX,1) is a 33 year old Belgian male. From the age of 17 he has complained of fatigue and intermittent pain in the neck and back muscles. He has mild scapular winging and mild weakness atrophy of several shoulder girdle muscles, and symmetric hypotrophy of the pectoral muscles. He has no complaints or clinical abnormalities in the lower limbs, no scoliosis or respiratory muscle weakness. Facial muscles are slightly hypotrophic and weak, without functional deficit. There is no cardiac involvement (normal ultrasound, ECG and Holter registration). His CK levels are high and variable (1600 -8700 U/L). A muscle biopsy at age 17 showed a mild dystrophy with a slight increase in fibers with one or more internal nuclei, necrotic and regenerating fibers, splitting fibers and mild type I muscle fiber predominance. Immunohistochemical stains for sarcolemmal and other proteins were normal, as was Western blotting for dystrophin and dysferlin.

The proband’s youngest sibling (IX,2), a 29 year old male, is less severely affected in a similar pattern and is still active as an amateur soccer player. CK levels are also elevated (1000 – 2300 U/L). A muscle biopsy at age 27 showed a moderate increase in fibers with one or more internal nuclei as the sole indication of a mild myopathy. Immunohistochemical stains for sarcolemmal and other proteins were normal, as was Western blotting for dystrophin and dysferlin.

No mutations had been detected in the CAPN3, FKRP, DMPK and ZNF9 genes; also Becker dystrophy and Facioscapulohumeral Dystrophy (FSHD) had been excluded.

We demonstrated that both the patients are compound heterozygous for two common mutations, c.191 dup A in exon 5 and p.Phe578Ser (c.1733 T>C) in exon 16.

Family X

The patient X,1 is an Italian male investigated at the age of 28 years, for the presence of increased CK levels (>3000 UI/L), not associated with muscle symptoms, except for cramps. The family screening for muscle disease revealed that also one of his sisters presented high CK levels, but not to the same degree. Muscle biopsy revealed a mild fiber size variability and immunoistochemistry a normal staining for dystrophin, sarcoglycans, calpain3 and dysferlin. Heart and respiratory function were normal. No other previous tests were referred. At the current age of 47 years, he remains asymptomatic except for myalgia and cramps. His elder sister (X,2) is still asymptomatic. We found in both of them c.191 dup A and a mutation p.Met839Arg (c.2516T>G) in exon 21.

Family XI

The proband, a 44-year-old Italian male patient, showed elevated C.K. levels (maximum 2200-3000 UI/l). A progressive atrophy and a weakness of biceps brachii muscles (asymmetric), hamstrings and hip adductors were detected on physical examination. Moreover, difficulties in climbing stairs were referred. In order to assess cardiac and respiratory function, Electrocardiogram (ECG), ultrasound and sprirometry were performed and cardiac or pulmonary involvement was excluded. A myopathic pattern was detected by Electromyography (EMG); myopathic changes and necrotic fibers were revealed by muscle biopsy. MRI of the lower limbs showed a replacement of gastrocnemius medialis and soleus. No mutations had been detected in DYSF gene.

ANO5 analysis showed c. 191 dup A and a novel nonsense mutation p.Gln421X (c.1261C>T, exon 13).

Family XII

The patient, a 71 year old French man, has developed since the age of 32 a distal lower leg weakness with the predominant involvement of triceps suralis muscles (MRC at the most recent examination: 2).

The weakness, slightly asymmetric, progressed to tibialis anterior and quadriceps muscles and to upper limb muscles involving the shoulder girdle, biceps muscles, and abdominal and axial muscles.

At the age of 55, a restrictive respiratory insufficiency was also detected at the follow up with FVC less than 60% and the patient was put in non-invasive ventilation. Mild dysphagia was noted and confirmed by videofluroscopic examination. The patient uses a bilateral cane to walk indoors and a manual wheelchair for long distances. Muscle biopsy showed abnormalities suggestive of a muscular dystrophy with a normal immunohystochemical and Western Blot expression of all the proteins involved in LGMD.

The patient did not disclose any results of previous molecular tests. NGS analysis permitted the identification of a homozygous missense mutation p.Gly231Val (c.692G>T) in exon 8.

Family XIII

The proband is an Italian 38 year old man, born to consanguineous healthy parents. He has complained since his thirties of diffuse myalgias, painful contractures and excessive fatigability. Neurological examination revealed a normal muscle strength and a mild left calf hypotrophy. Serum CK levels were constantly elevated ranging from 1500 up to 3000 IU/L. Electromyography (EMG) was unremarkable. He underwent two muscle biopsies in 2006 and 2011 respectively and in both only unspecific minimal changes were present with increased muscle fiber variability, centralized nuclei and some hypotrophic fibers. Dystrophin, sarcoglycan, caveolin, dysferlin expression was normal as well as calpain expression. Biochemical analysis on muscle homogenate did not reveal any enzymatic defect. Muscle MRI showed no significant changes at the muscles limbs. Cardiac and respiratory functions were normal.

No other previous tests were shown. NGS screening detected c.191 dup A in homozygosity.

Family XIV

The proband (XIV,1) is an Italian 45 year old man, who, from the age of about 40 years, has started to complain of muscle pain and cramps in particular in the calves. A constant high level of blood CK (> ​​1000) was recorded with a normal neurological exam, except for a slight salience of calves. He underwent an EMG exam, that was normal, and a muscle biopsy that showed mild myopathic changes with normal dystrophin, sarcoglycans, caveolin, dysferlin and spectrin expression. Heart, lung and cognitive functions were normal. Mutations in dysferlin gene had been excluded by a previous test. By NGS strategy, we detected the homozygous c.1627dupA in the exon 20.

A brother (XIV,2), a 38 year old man, showing constant elevations of CK (> ​​1000), but still asymptomatic was also found to be mutated.

Family XIX

The proband was born from consanguineous parents. One cousin of the mother was bedridden since age 18 years. The mother of the patient is referred to be affected. His 12 year-old daughter has mildly elevated CK levels (250U/L, n.v. 24-170).

Age at onset 44 years with easy fatigability and difficulty walking. He had an unspecified liver disorder with elevated transaminases and myoglobinemia. CK levels were found elevated, ranging from 1700-3450 U/L. Because of muscle weakness, a first muscle biopsy (tibialis anterior muscle) was performed in another center and was interpreted as inflammatory myopathy and a steroid therapy was offered. Muscle CT scan showed atrophy of adductor magnus and biceps femoris. Muscle MRI showed fatty degeneration in the legs (adductors and biceps femoris), and oedema of gracilis muscle.

At age 45 years, EMG was myogenic. He complained of pain in legs, knees, heels, back, and upper girdle muscles. On neurological examination he presented mild-moderate weakness in distal and proximal muscles of the upper and lower girdles. He presents tremors that assume the characteristic of rippling muscle phenomenon in the quadriceps femoris.

Sequencing of CAV3 and RYR1 genes was negative.

A second muscle biopsy at age 45 years revealed dystrophic features with fibers undergoing phagocytosis and regeneration, and increased central nuclei. Normal protein expression for dystrophin, dysferlin, calpain-3, alpha-sarcoglycan, caveolin-3, emerin, alpha-dystroglycan was found. CK was 2272 U/L.
